# Discovery of vanoxerine dihydrochloride as a CDK2/4/6 triple-inhibitor for the treatment of human hepatocellular carcinoma

**DOI:** 10.1186/s10020-021-00269-4

**Published:** 2021-02-12

**Authors:** Ying Zhu, Kun-Bin Ke, Zhong-Kun Xia, Hong-Jian Li, Rong Su, Chao Dong, Feng-Mei Zhou, Lin Wang, Rong Chen, Shi-Guo Wu, Hui Zhao, Peng Gu, Kwong-Sak Leung, Man-Hon Wong, Gang Lu, Jian-Ying Zhang, Bing-Hua Jiang, Jian-Ge Qiu, Xi-Nan Shi, Marie Chia-mi Lin

**Affiliations:** 1 Biomedical Engineering Research Center, Kunming Medical University, Kunming, 650500 Yunnan China; 2 Department of Pathology, Yunnan University of Chinese Medicine, Kunming, 650504 Yunnan China; 3 Department of Cadre Medical Branch, The 3rd Affiliated Hospital of Kunming Medical University, Kunming, 650118 Yunnan China; 4 Academy of Medical Science, Zhengzhou University, Zhengzhou, 450000 Henan China; 5 Department ofMedicine, Southwest Guizhou Vocational and Technical College for Nationalities, Xingyi, 562400 Guizhou China; 6 CUHK-SDU Joint Laboratory On Reproductive Genetics, School of Biomedical Sciences, The Chinese University of Hong Kong, Hong Kong, China; 7 Department of the Second Medical Oncology, The 3rd Affiliated Hospital of Kunming Medical University, Yunnan Tumor Hospital, Kunming, 650000 China; 8 Department of Urology, The 1st Affiliated Hospital of Kunming Medical University, Kunming, 650000 China; 9 Department of Geriatric Cardiology, The 1st Affiliated Hospital of Kunming Medical University, Kunming, 650000 China; 10 Department of Teaching and Research of Formulas of Chinese Medicine, Yunnan University of Chinese Medicine, Kunming, 650000 Yunnan China; 11 Department of Computer Science and Engineering, The Chinese University of Hong Kong, Hong Kong, 999077 China; 12 Department of Physiology, Yunnan University of Chinese Medicine, Kunming, 650504 Yunnan China

**Keywords:** Cyclin-dependent kinases 2/4/6, Hepatocellular carcinoma, Vanoxerine dihydrochloride, Triple inhibitor, Drug combination

## Abstract

**Background:**

Cyclin-dependent kinases 2/4/6 (CDK2/4/6) play critical roles in cell cycle progression, and their deregulations are hallmarks of hepatocellular carcinoma (HCC).

**Methods:**

We used the combination of computational and experimental approaches to discover a CDK2/4/6 triple-inhibitor from FDA approved small-molecule drugs for the treatment of HCC.

**Results:**

We identified vanoxerine dihydrochloride as a new CDK2/4/6 inhibitor, and a strong cytotoxicdrugin human HCC QGY7703 and Huh7 cells (IC50: 3.79 μM for QGY7703and 4.04 μM for Huh7 cells). In QGY7703 and Huh7 cells, vanoxerine dihydrochloride treatment caused G1-arrest, induced apoptosis, and reduced the expressions of CDK2/4/6, cyclin D/E, retinoblastoma protein (Rb), as well as the phosphorylation of CDK2/4/6 and Rb. Drug combination study indicated that vanoxerine dihydrochloride and 5-Fu produced synergistic cytotoxicity in vitro in Huh7 cells. Finally, in vivo study in BALB/C nude mice subcutaneously xenografted with Huh7 cells, vanoxerine dihydrochloride (40 mg/kg, i.p.) injection for 21 days produced significant anti-tumor activity (p < 0.05), which was comparable to that achieved by 5-Fu (10 mg/kg, i.p.), with the combination treatment resulted in synergistic effect. Immunohistochemistry staining of the tumor tissues also revealed significantly reduced expressions of Rb and CDK2/4/6in vanoxerinedihydrochloride treatment group.

**Conclusions:**

The present study isthe first report identifying a new CDK2/4/6 triple inhibitor vanoxerine dihydrochloride, and demonstrated that this drug represents a novel therapeutic strategy for HCC treatment.

## Introduction

Hepatocellular carcinoma (HCC), the most common type of liver cancer, is the second (Mazzanti et al. 2016) cause of cancer-related death world-wide. Surgical resection is the first line treatment, followed by liver transplantation and percutaneous ablation. There is a high frequency of tumor recurrence after surgical resection, and most HCCs are resistant to conventional chemotherapy and radiotherapy (Llovet et al. 2015). Emerging targeted therapies have provided new treatment options (Jindal et al. 2019), with sorafenib, a multitarget tyrosine kinase inhibitor (TKI) approved by FDA for the treatment of unrespectable HCC. However, rapid development of drug resistance limited the uses. There is urgent need for the development of more effective drugs targeting different mechanisms.

Cyclin-dependent kinases (CDKs) are important targets for cancer therapy, as they play critical roles in cell cycle and cell growth/differentiations (Lim and Kaldis 2013; Asghar et al. 2015). There are two categories of CDKs. The first category, includes CDK1,CDK2,CDK4andCDK6, regulates cell cycle progression from G0 phase to G1, and S phases (Hinz et al. 1999; Lukas et al. 1996). The second category, includes CDK7-11 and CDK14-20, regulates gene transcription (Peng et al. 1998; Nemet et al. 2014) and Wnt signaling (Sun et al. 2014). CDK inhibitors have long been evaluated as cancer therapeutics (Asghar et al. 2015; Canavese et al. 2012; Peyressatre et al. 2015; Shen et al. 2019).Currently, the third generation CDK4/6 dual inhibitors palbociclib, ribociclib, and abemaciclib have been approved by FDA for the treatment of breast cancer (Spring et al. 2019). Unfortunately, they have only limited benefits for the treatment of HCC or other cancers, suggesting the need for the development of more effective CDK inhibitors.

It has been demonstrated that the dysregulation of any one of the CDK2/4/6 is sufficient to cause HCC. For example, transgenic mice overexpressing either CDK2 or CDK4 or CDK6, all led to the development of liver cancer (Otto and Sicinski 2017). Furthermore, significantly elevated expressions of CDK2, CDK4, and CDK6 are well documented in HCC and many other cancers (Li et al. 2002; Kim et al. 2000; Che et al. 2012; Yamamoto et al. 1995), and they are the causal factors for the development and progression of cancer. Therefore, a CDK2/4/6 triple-inhibitor appears to be a logical strategy for the treatment of HCC and many other cancers.

We have previously reported the identification of two CDK2 inhibitors, adapaline and fluspirilene and one CDK4/6 dual inhibitor rafoxanide (Shi et al. 2015a,2015b,^2018^) by the combination of computer-aided strategies and the experimental validations. In this study, we extended these strategies to screen for CDK2/4/6 triple inhibitors from FDA approved drugs, and discovered vanoxerine dihydrochloride as a new CDK2/4/6 triple inhibitor for the treatment of HCC. Results from this study demonstrated that vanoxerine dihydrochloride exhibited strong cytotoxic effect in human HCC QGY7703 and Huh7 cells. It caused G1-arrest, induced apoptosis, and reduced the expressions of CDK2/4/6, cyclin D/E, retinoblastoma protein (Rb). We also validated its efficacy in vivo in BALB/C nude mice xenografted subcutaneously with Huh7 cells. The anti-tumor activity of vanoxerine dihydrochloride was comparable to that achieved by 5-Fu. Furthermore, combined administration of vanoxerine dihydrochloride and 5-Fuproduced synergistic effect.

To our knowledge, this is the first report identifying a CDK2/4/6 triple inhibitor. As a FDA approved drug, the potential use of vanoxerine dihydrochloride for the treatment of HCC warrants further investigations.

## Materials and methods

### Computer-aided structure-based virtual screening for CDK2/4/6 triple inhibitors

The chemical structures of a total of 3167 US Food and Drug Administration (FDA) approved drugs were gathered from the ZINC database (Irwin and Shoichet 2005; Irwin et al. 2012). The X-ray crystallographic protein structures used in the studies were obtained from the Protein Data Bank (PDB) (Berman et al. 2000; Huang and Wong 2016).We selected 5 structures of CDK4 and 8 structures of CDK6, and first screened for CDK4/6 inhibitors as described in our previous studies (Shi et al. 2015b,^2018^). The co-crystallized ligands and water molecules were manually removed. The free and open-source docking docking software idock v2.2.1 developed by our group (Li et al. 2014,2012) was used to dock all of the compounds onto all of the ATP binding pocket of all of the CDK4/6 structures using an ensemble docking strategy, to predict their binding conformations and binding affinities as described in previous study (Shi et al. 2018). For each compound, idock outputted nine predicted conformations, and the conformation with the best idock score was selected. The 3167 compounds were sorted in the ascending order of their predicted binding free energy averaged across the 13 CDK4/6 structures, and the top-scoring 50 candidate CDK4/6 inhibitors were visually examined using iview. We also collected 14 structures of CDK2 from the 44 CDK2 structures we examined in the previous studies (Shi et al. 2015a,b). These top 50 candidate inhibitors were then docked onto all of the ATP binding pocket of CDK2 structures. The high-scoring drugs were manually examined based on molecular weight and other drug-like properties. Nine top ranking commercially available compounds were purchased and evaluated.

## Chemicals

Monatepil, Fluazuron, Temafloxacin, Ketanserin, Talniflumate, Altanserin, Dutasteride, Mizolastine, Vanoxerine dihydrochloride, 5-Fu were purchased from Sigma-Aldrich.

### Cell lines, cell culture, and experimental conditions

The human HCC cell lines QGY7703 and Huh7 were obtained from Cell bank of Chinese Academy of Sciences(Shanghai, China), and cultured in D-MEM/F-12 medium (GIBCO, USA) containing 8% FBS (Hyclone, Mexico) at 37 °C in 5% CO2 and 95% humidified air. Cells were plated in 96-, 24-plates (NEST, China) with medium containing 8% FBS and the test compounds at indicated concentrations (1, 3, 10 and 30 μM), and incubated for indicated times (6, 12, 24, 48 or 72 h).

### Cell viability MTT and CCK-8 assays

MTT assay were conducted as described in previous studies (Shi et al. 2015a,b,^2018^).QGY7703 and Huh7 cells were plated at an initial density of 9 × 10^3^ cells/well in 96-well plates, incubated with MTT (Sigma) reagents and the absorbance measured at 570 nm with a microplate reader (Multiskan Spectrum, Thermo Scientific *Microplate Reader*, USA). CCK-8 assay was performed as described in the CCK-8 Kit (Dojindo Laboratories). Cells were seeded in 96-well plate, treated with various drugs for indicated time prior to the addition of CCK-8 solution and OD values were measured at 450 nm using a microplate reader.

### Cell cycle analysis

The cell cycle profile was determined by Flow cytometry analysis, as described previously (Shi et al. 2015a,b,^2018^).Briefly, QGY7703 and Huh7 cells (4 × 10^4^) were seeded in 24-well plates in D-MEM/F-12 medium. After 24 h culture, medium were replaced with D-MEM/F-12 containing 8% FBS and vanoxerine dihydrochloride (1, 3, 10 or 30 μM) and incubated at 37 °C for indicated times (6, 12 or 24 h). At the end of experiment, cells were fixed in ice-cold ethanol, and stained in Coulter DNA-Prep Reagents (Beyotime Coulter, BeyotimeInstitute of Biotechnology, Beijing). The cellular DNA content was determined by EPICS xL4 flow cytometer (BDFACSCalibu, USA), and cell cycle distribution determined by BD FACStation software (USA).

### Cell apoptosis

QGY7703 and Huh7 cells were seeded in 6-well platein D-MEM/F-12 medium. After 48 h culture, the medium were replaced with D-MEM/F-12 containing 8% FBS and various concentrations of vanoxerine dihydrochloride (1, 3, 10 or 30 μM), and incubated at 37 °C for indicated times (6, 12 or 24 h). Apoptosis was measured by annexinV and propidium iodide (PI) staining (Beyotime Institute of Biotechnology, Beijing) as described in previous studies (Shi et al. 2015a,b,^2018^).

### Western blot analysis

Cells were lysed and Western blotting analysis were performed as described previously (Shi et al. 2015a,b,^2018^).QHY7703 and Huh7 cells were plated at 6-well plates, cultured in serum starved media (0.125% FBS) at 37 °C for 24 h, and then with 10%FBS medium containing various concentrations (3, 10, 30 μM) of vanoxerine dihydrochloride. Cells were harvested after 6 h incubation and proteins analyzed by Western blotting. Primary antibodies were purchased from Cell Signaling Technology, Inc. Danvers, MA, USA). They include anti-cyclin D1 (no. 2978), anti-cyclinE (no. 4129), anti-CDK2/4/6 (no. 2546), anti-Rb (no. 9313), anti-phospho-CDK4, anti-phospho-CDK2/4/6 (no. 2561), anti-Rb (no. 9301), and anti-GAPDH (no. 5174). As positive controls, three siRNAs targeting each of the CDK2/4/6were designed as described previously (Shi et al. 2018), and used to inhibit the expressions of each of the CDK2/4/6proteins in QGY7703 and Huh7 cells. The proteins were measured using enhanced chemiluminescence detection system (Thermo Fisher scientific, USA).

### Synergy quantitation of the drug combination study

Synergy quantitation of the drug combination studies were performed according to the Chou–Talalay method. Huh7 cells were plated at an initial density of 5 × 10^3^ cells/well in 96-well plates, and cells were treated with various concentrations of vanoxerine dihydrochloride and 5-Fu. After 72 h treatment, cell viability was determined by CCK-8 assay and the absorbance values were measured at 450 nm using microplate reader. The combined effect was analyzed by CompuSyn software (www.combosyn. com), which performs multiple drug dose–effect calculations using the Median Effects methods described by Chou and Talalay to determine the combination index (CI). The drug combinations quantitative definitions of CI are (1) CI = 1 represents additive effect, (2) CI < 1 represents synergistic effect, and (3) CI > 1 represents antagonism. The formula of the combined index of the two drugs is: CI = (D)1/(Dx)1 + (D)2/(Dx)2, Single dose (D), combined dose (Dx)) (Wang et al. 2013).

### *Ethic statement and the *in vivo* nude mice xenograftedstudy*

The animal studies were approved by the Kunming Medical University’s laboratory animal ethics committee. Female BALB/C nude mice (4–5 weeks old, weighing 15 g; Vital River Laboratory Technology Co. Ltd., Beijing, China), were housed and cared under standard conditions (pathogen-free, 12 h light/dark cycle, 50–80% humidity, and 15–27 °C) in accordance with guidelines from animal ethics committee in Kunming Medical University. To establish the xenografted model in nude mice (n = 20),Huh7 cells (1 × 10^6^ in 0.2 ml PBS) were subcutaneously injected into the right flank, and tumor size measured daily. At seven days after inoculation (tumor volume 80–100 m^3^), mice were divided randomly to four groups (5 mice/group) and given daily intraperitoneal injection of (1) vanoxerine dihydrochloride (40 mg/kg), (2) 5-Fu (10 mg/kg), (3) vanoxerine dihydrochloride (40 mg/kg) plus 5-Fu (10 mg/kg), (4) control PBS, for 21 days. At the end of experiments, mice were sacrificed by cervical dislocation, tumors excised, weighed, images captured, and immunohistochemistry analysis performed. The tumor volume was calculated by V = ab2/2 (a = longest axis; b = shortest axis).

### Immunohistochemistry

Tumor tissues were fixed in 10% formalin and embedded in paraffin, sliced into 4 μm sections, deparaffinized, dehydrated, antigen retrieved, blocked with 5% goat serum, and incubated in the primary antibodies: anti-RB1 (1: 500; CST), anti-CDK2 (1:50; Abcam), anti-CDK4 (1: 500; CST), anti-CDK6 (1: 100 Abcam). The slides were washed and incubated with biotinylated anti-mouse or anti-rabbit secondary antibodies. The peroxidase reaction was visualized using 3,3′-diaminobenzidine tetrahydrochloride (DAB) and counterstained with hematoxylin. To quantitate the staining intensity, 5 random fields were chosen, and the numbers of total cells and positive cells were counted in each section under a microscope at 400 × magnification. The percentage of positive cell populations from the 5 random fields was analyzed for statistics.

### Statistical analysis

Data were obtained from the triplicates of three different experiments. Values are expressed as the mean ± standard deviation. The dates were analyzed by SPSS software (version 16.0). P < 0.05 was considered to indicate statistically significant difference between values.

## Results

### *Discovery of CDK2/4/6 triple-inhibitors *via* computer-aided structure-based virtual screening*

The chemical structures of a total of 3167 US Food and Drug Administration (FDA) approved drugs were gathered from the ZINC database, first docked onto the CDK4 and CDK6 structures, and then sorted in the ascending order of their predicted binding free energy. The top 50 ranking candidate CDK4/6 inhibitors were then docked onto CDK2 structures to screen for CDK2/4/6 triple inhibitors. We manually examined the high-scoring compounds based on in silico estimations of binding strength, appropriate molecular weight and other drug-like properties, and complementary matching of molecular shape. The highest-scoring compounds were identified and nine commercially available compounds (Table 1) were selected for subsequent validations (Ikeno et al. 1998; Bull et al. 1996; Chin et al. 1988; Nueten et al. 1981; Donnelly and Rogers 2003; Sietnieks 1985; Bramson et al. 1997; Danjou et al. 1992; Nagase et al. 1987a).

**Table 1:** The nineteen top-scoring compounds purchased and tested in vitro

Compounds name	ZINC ID	Average idock score(kcal/mol)	MW (g/Mol)	Clinic usage	Refs
Monatepil	1,851,142	− 9.57	475.62	Ca2 + channel antagonist	Ikeno et al. (1998)
Fluazuron	2,570,819	− 10.05	506.21	Insecticides	Bull et al. (1996)
Temafloxacin	9,133,461	− 9.58	417.38	Difluoro quinolone antimicrobial agent	Chin et al. (1988)
Ketanserin	537,877	− 9.36	545.51	A selective 5-HT2 receptor antagonist	Nueten et al. 1981)
Talniflumate	1,844,627	− 10.08	414.33	Inhibitor of humancalcium-activated chloride channels	Donnelly and Rogers (2003)
Altanserin	26,174,383	− 9.58	411.49	The selective 5-hydroxytryptamine2 (5-HT2) receptor antagonist	Sietnieks 1985)
Dutasteride	3,932,831	− 9.58	528.53	Selective inhibition of type 2 5alpha-reductase	Bramson et al. 1997)
Mizolastine	13,831,810	− 9.48	432.49	Selective H1-receptor blocker	Danjou et al. (1992)
Vanoxerine dihydrochloride	22,034,135	− 8.83	523.49	Inhibitor of uptake of dopamine and norepinephrine	Nagase et al. (1987a)

An idock score is the estimated binding free energy (kcal/mol units). Negative value implies a high predicted binding affinity

### The cytotoxicity of candidate drugs on human HCC QGY7703 and Huh7 cells

**Fig. 1:**
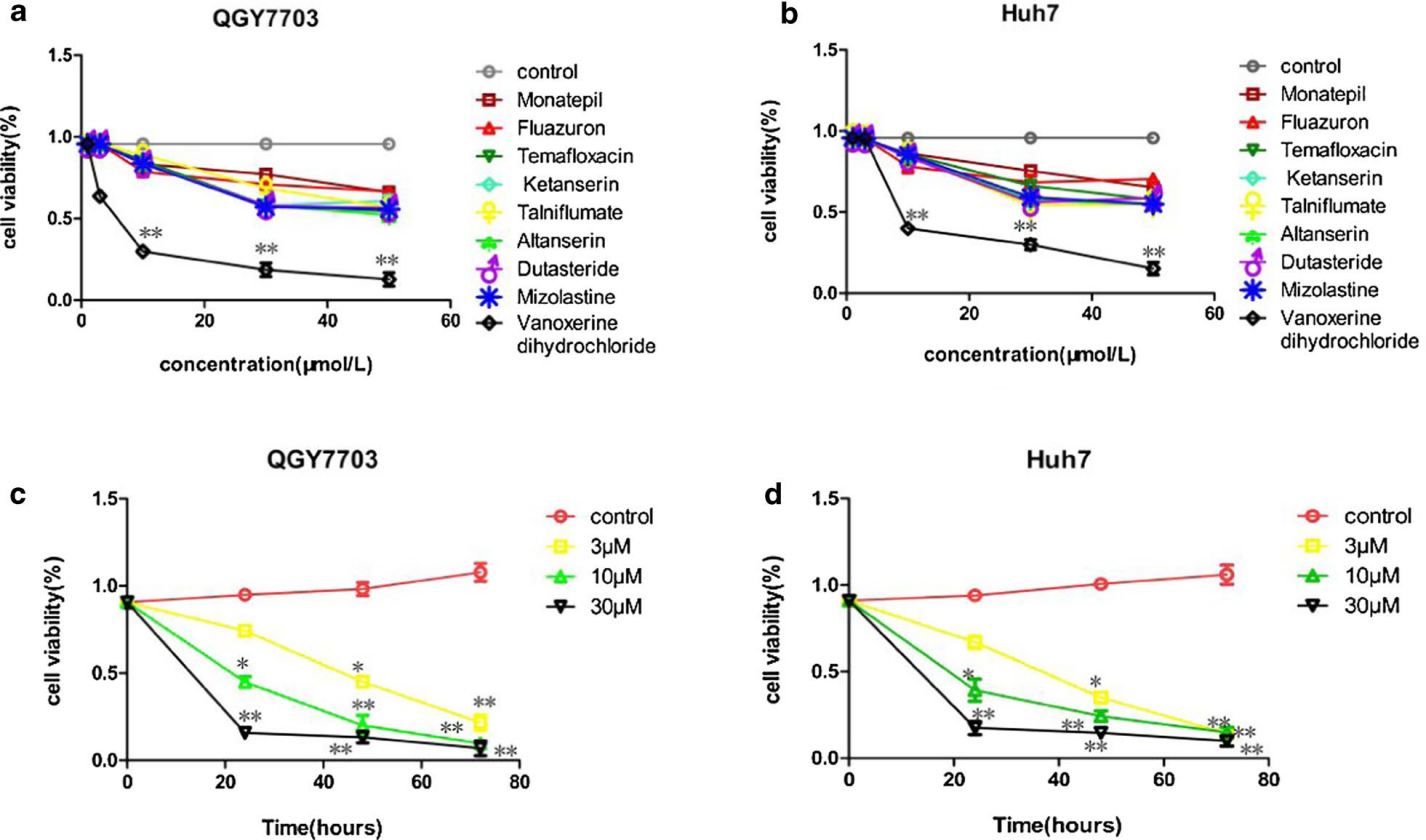
The effect of nine candidate CDK4/6 inhibitors on the viability of QGY7703 and Huh7 cells. The effect of nine candidate compounds on the cell viability of **a** QGY7703 and **b** Huh7 cell lines as determined by MTT assay. Vanoxerine dihydrochloride exhibited the highest cyto-toxicity in both cell lines. Vanoxerine dihydrochloride reduced cell viability dose- and time-dependently in **c** QGY7703 and **d** Huh7 cell lines. IC50 values was calculated to be 3.79 μM for QGY7703 and 4.04 μM for Huh7. *p < 0.05, significantly different from the control PBS treatment group

We first evaluated the effects of these nine compounds (monatepil、fluazuron、temafloxacin、KETANSERIN、talniflumate、altanserin、dutasteride、mizolastine、vanoxerine dihydrochloride) on reducing cell viability, as determined by MTT assay. These compounds caused reduced cell viability in QGY7703 (Fig. 1a) and Huh7 cells (Fig. 1b), with vanoxerine dihydrochloride most effective. Furthermore, the inhibitory effect of vanoxerine dihydrochloride was dose- and time-dependent (Fig. 1c, d), with the IC50 values calculated (using GraphPad Prism5) to be 3.79 μM for QGY7703 and 4.04 μM for Huh7 cells.

### Vanoxerinedihydrochloride treatment caused cell cycle arrest and apoptosis in QGY7703 and Huh7 cells

**Fig. 2:**
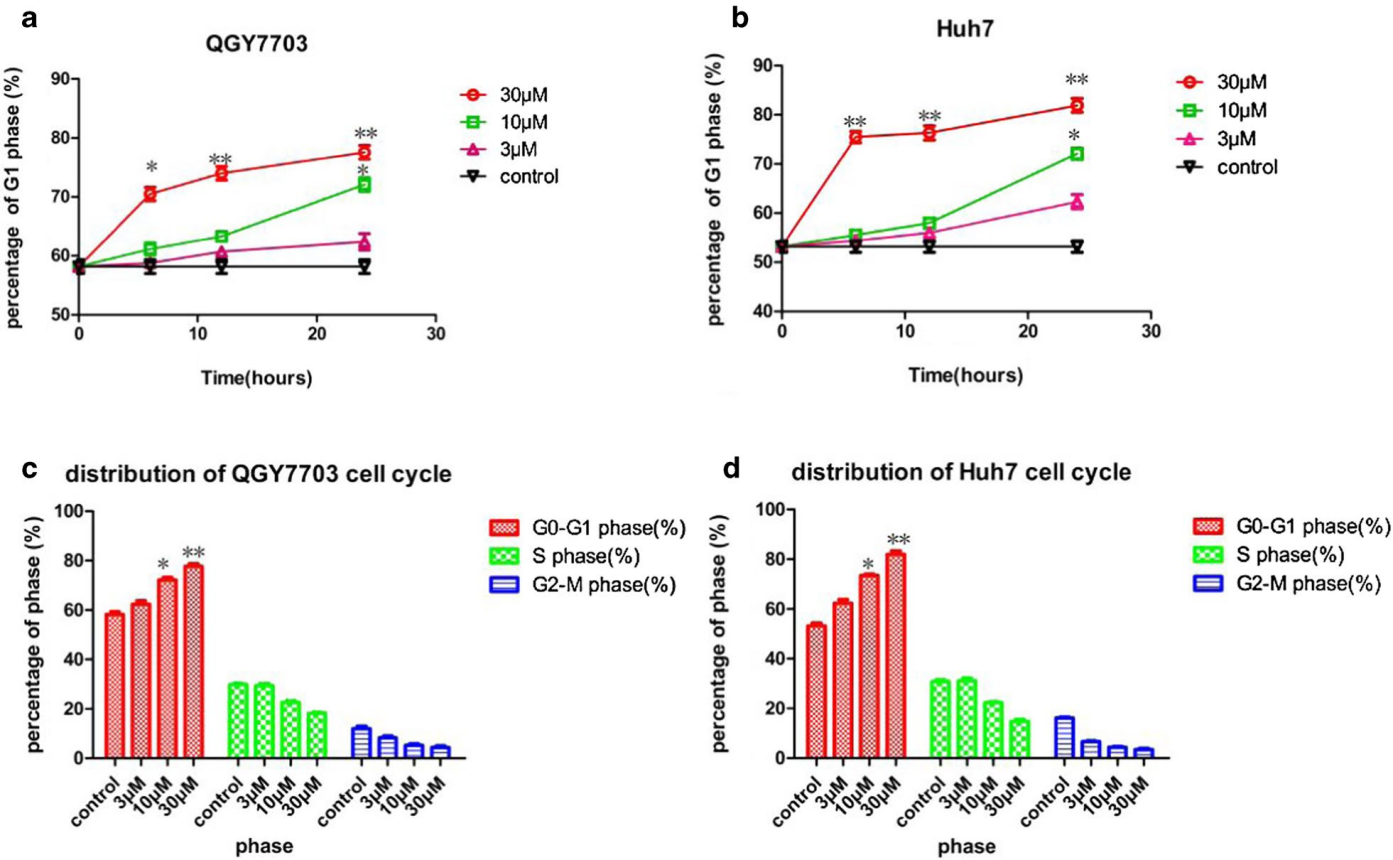
Effect of vanoxerine dihydrochloride treatment on cell cycle distribution in QGY7703 and Huh7 cells. **a** QGY7703 and **b** Huh7 cells were treated with different concentrations (3, 10 and 30 μM) of vanoxerine dihydrochloride for 6, 12, 24 hours. Cell cycle distributions were measured by flow cytometry. Vanoxerine dihydrochloride dose- and time-dependently increased the % of cells in G1 phase, as compared to PBS control. **b** The cell cycle distributions at 24 h after 10 μM vanoxerine dihydrochloride treatment. The bar graph indicated the percentage of the G1, S and G2-M phases. *p < 0.05, significantly different from the control PBS treatment group

**Fig. 3:**
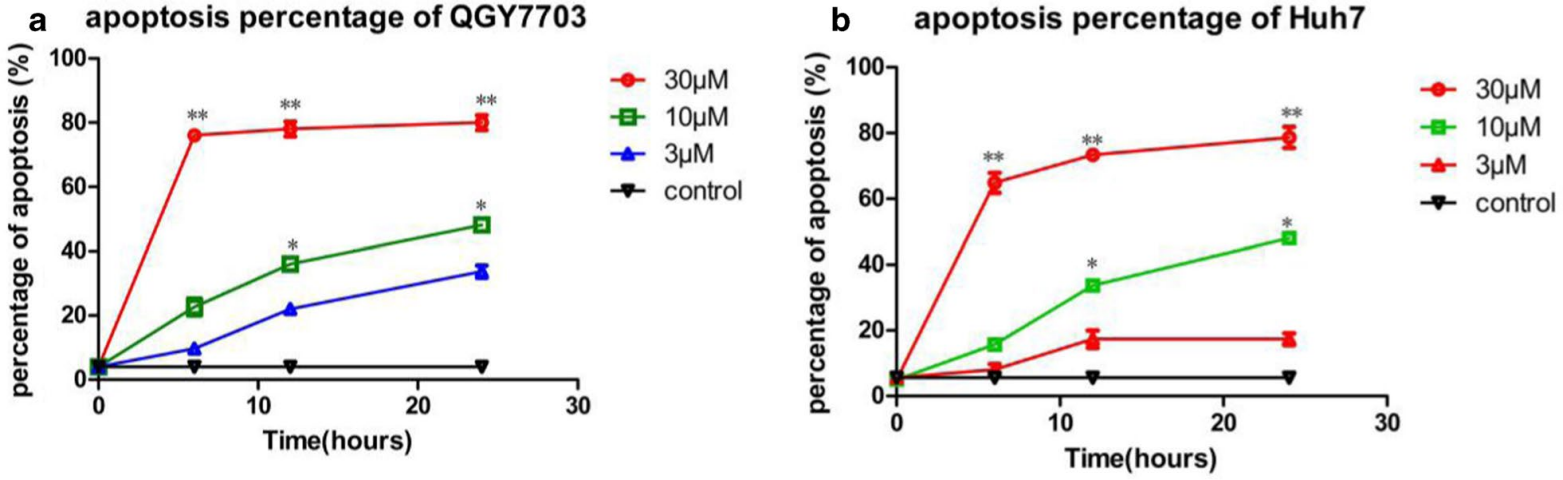
Vanoxerine dihydrochloride treatment induced cell apoptosis. Vanoxerine dihydrochloride treatment at at 3, 10, 30μMconcentration for 6, 12, 24 h significantly increased the percentage of apoptosis in **a** QGY7703 and **b** Huh7 cell lines in a dose-and time-dependent manner. *p < 0.05, significantly different from the control PBS treatment group

To demonstrate that vanoxerine dihydrochloride is a CDK2/4/6 triple inhibitor, we treated QGY7703 and Huh7 cells with vanoxerine dihydrochloride (3, 10 or 30 μM) for 6, 12 or 24 h, and determined its effects on the cell cycle profiles, using flow cytometry an analysis. As shown in Fig. 2, vanoxerine dihydrochloride treatment significantly (p < 0.05) caused the G1-phase arrest in a dose-and time-dependent manner in QGY7703 (Fig. 2a) and Huh7 (Fig. 2b) cells. Significantly decreased cell populations in the S-phase and G2-M phase were also observed in QGY7703 (Fig. 2c) and Huh7 (Fig. 2d) cells at 24 h after treatment. In addition, we also showed that vanoxerine dihydrochloride treatment significantly promoted cell apoptosis, as determined by flowcytometry analysis using the annexinV and propidium iodide staining. Vanoxerine dihydrochloride treatment (at 3, 10, 30 μM for 6, 12, 24 h) significantly increased the percentage of apoptotic cells in a dose-and time-dependent manner in QGY7703 (Fig. 3a) and Huh7 (Fig. 3b) cells.

### Vanoxerine dihydrochloride decreased the expressions and phosphorylations of CDK2/4/6

**Fig. 4:**
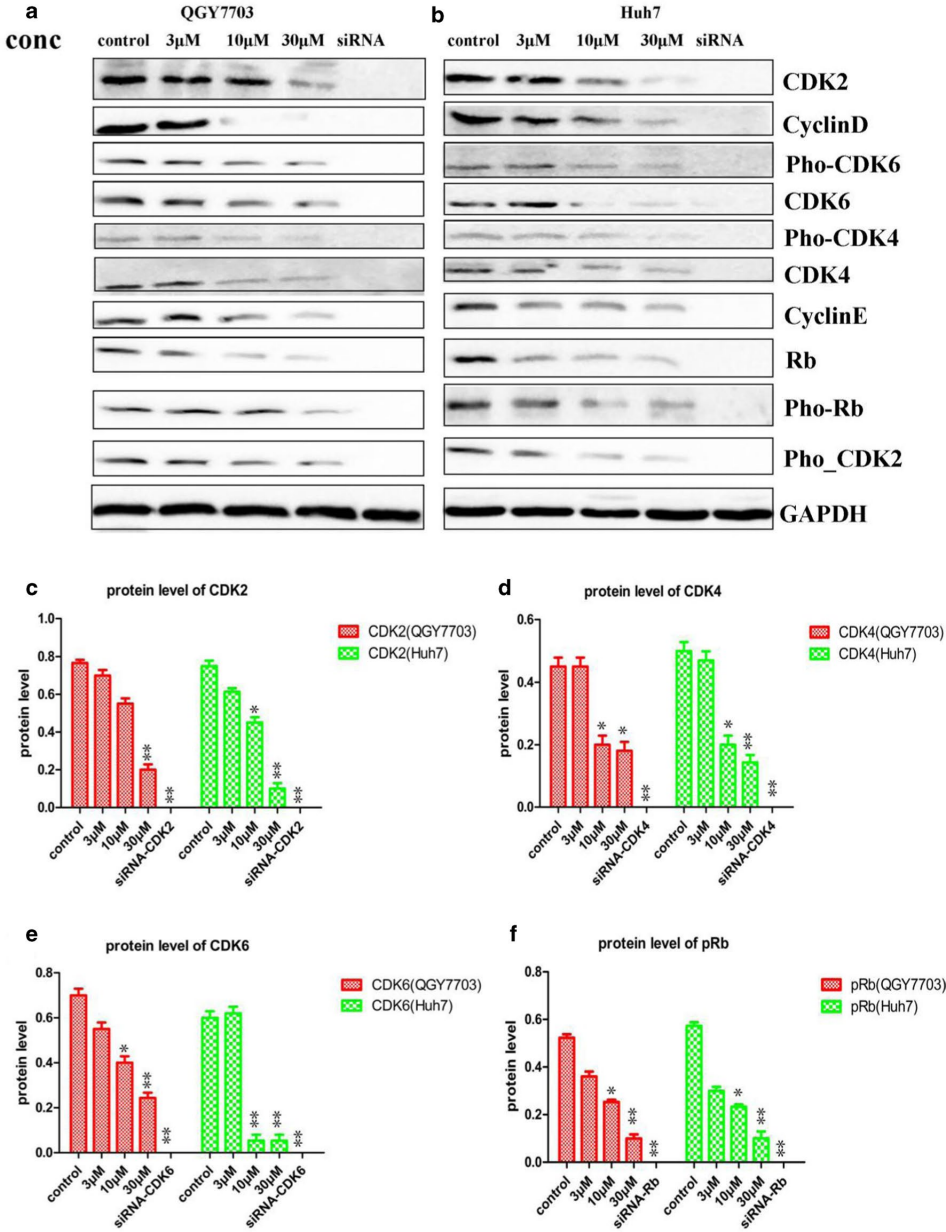
Effects of Vanoxerine dihydrochloride treatment on the expressions of proteins that play key roles in cell cycle progressions. **a** QHY7703 and **b** Huh7 cells were plated at 6-well plates with 0.125% FBS medium for 24 h and then with 10% FBS medium containing various concentrations (3, 10, 30 μM) of vanoxerine dihydrochloride. Cells were harvested after 6 h incubation and proteins analyzed by Western blotting. Western blotting results showed that vanoxerine dihydrochloride treatment significantly reduced the expressions of CDK2/4/6, Rb, pho-CDK2/4/6, pho-Rb and cyclin D/E in QGY7703 and Huh7 cells. As positive controls, three siRNAs targeting each of the CDK2/4/6 were designed as described previously (Shi et al. 2018), and used to inhibit the expressions of each of the CDK2/4/6proteins. *p < 0.05, significantly different from the control PBS treatment group

**Fig. 5:**
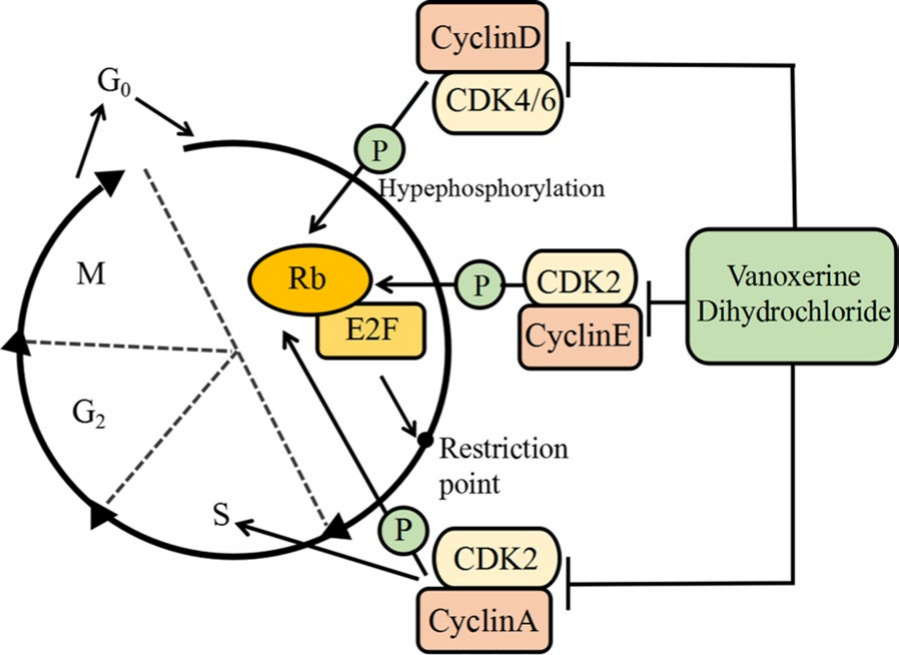
Mechanisms of vanoxerine dihydrochloride. Vanoxerine dihydrochloride inhibited CDK4/6 phosphorylation and the complex with cyclinD. It also inhibited CDK2 phosphorylation and the complex with cyclinE. Together, they suppress the hyperphosphorylation of RB, and the releases of pRB from its association with transcription factor E2F. As a result, it inhibits the cell cycle to proceed from G1 to S-phase. In addition, vanoxerine dihydrochloride also reduced cyclinA-CDK2 complex, and inhibited DNA replication and decrease S and G2-M phases

Western blotting analysis was used to measure the effects of vanoxerine dihydrochloride treatment on the expressions and phosphorylations of CDK2/4/6, the downstream target protein Rb, and their binding partners cyclinD/E, in QGY7703 and Huh7 cells. As expected of a CDK2/4/6 triple inhibitor, vanoxerine dihydrochloride significantly and dose-dependently decreased the expressions of CDK2/4/6, the pho-CDK2/4/6, the binding partners cyclinE and cyclinD, as well as the down-stream target proteins Rb and pho-Rb in QGY7703 (Fig. 4a) and Huh7 (Fig. 4b) cells. In summary, we proposed the molecular mechanisms of vanoxerine dihydrochloride (Fig. 5), in which vanoxerine dihydrochloride inhibited CDK4/6 phosphorylation, which reduced the complex of cyclin D and CDK4/6. As a CDK2 inhibitor, it also inhibited CDK2 phosphorylation, which reduced the complex of cyclinE-CDK2. Together, they caused the subsequent reduction of Rb phosphorylation as well as the activation of E2F, to inhibit G1-S transition and produce G1 arrest. In addition, it is also expected to suppress the activation of cyclinA-CDK2 complex to decrease DNA replication and cell cycle S to G2-M phase transitions, which is consistent with what we observed from the cell cycle profiles analysis.

### The predicted conformations of vanoxerinedihydrochloride and CDK2/4/6

**Fig. 6:**
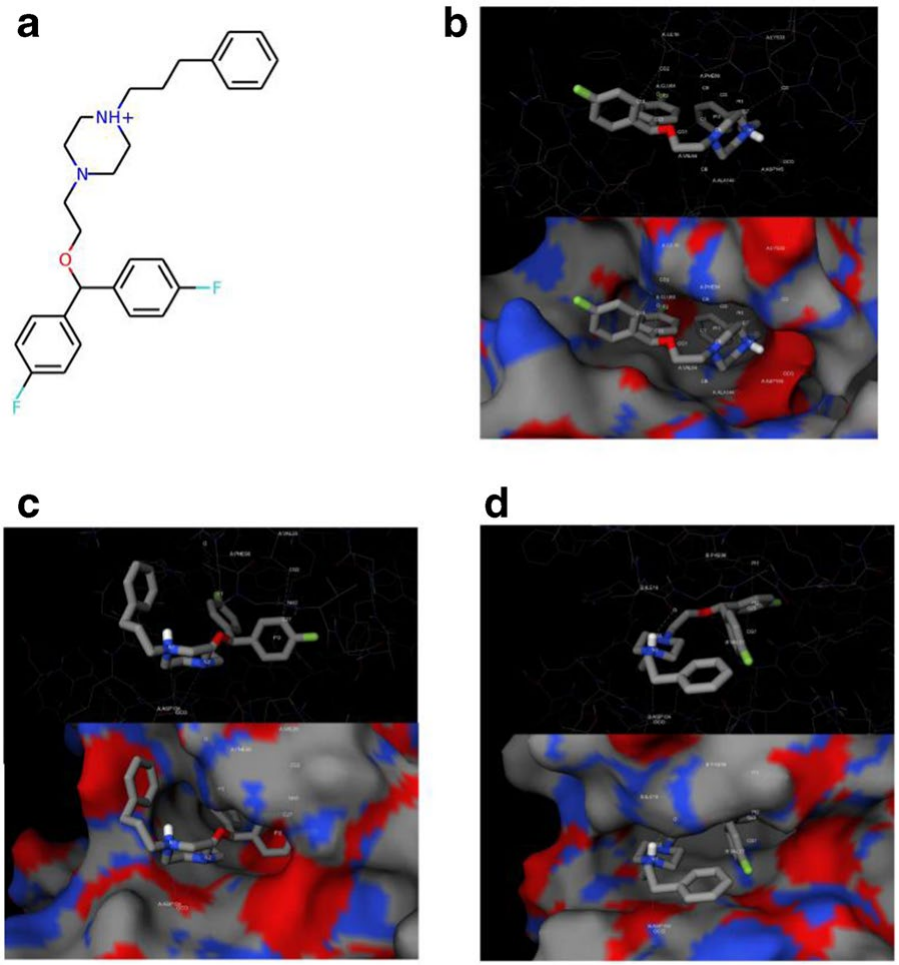
Structural analysis of the predicted conformation of vanoxerinedihydrochloride in CDK2/4/6 revealed critical binding interactions. **a** The depicted two-dimensional structure of vanoxerinedihydrochloride. The predicted binding conformation in complex with **b** CDK2, **c** CDK4, and **d** CDK6. According to the docking result, vanoxerinedihydrochloride binds to CDK2 through hydrophobic contacts with ILE10, LYS33, VAL64, PHE80 and ALA144, a salt bridge with ASP145 and a halogen bond with GLU81. It interacts with CDK4 through two salt bridges with ASP104, a π interaction with LYS40, and a halogen bond with PHE98. It binds to CDK6 through a hydrogen bond with ILE19, a salt bridge with ASP104 and a π interaction with PHE98

The predicted two-dimensional chemical structure of vanoxerinedihydrochloride is shown in Fig. 6a. Based on the results from computer docking, we predicted that vanoxerinedihydrochloride interacts with CDK2 and resides in the ATP-binding site of CDK2 with hydrophobic binding with ILE10, LYS33, VAL64, PHE80, ALA144, and a salt bridge with ASP145, and a halogen bond with GLU81 (Fig. 6b). It interacts with CDK4 ATP-binding site through two salt bridges with ASP104, a π interaction with LYS40, and a halogen bond with PHE98 (Fig. 6c), and interacts with CDK6 ATP binding site through a hydrogen bond with ILE19, a salt bridge with ASP104 and a π interaction with PHE98 (Fig. 6d). Results from western blotting indicated that vanoxerine dihydrochloride inhibited the activities of CDK2/4/6 with similar efficacy, suggesting that it has comparable binding affinity to all three CDKs.

### *Vanoxerine dihydrochloride and 5-FU produced synergistic cytotoxic effects *in vitro* in Huh7 cells*

**Fig. 7:**
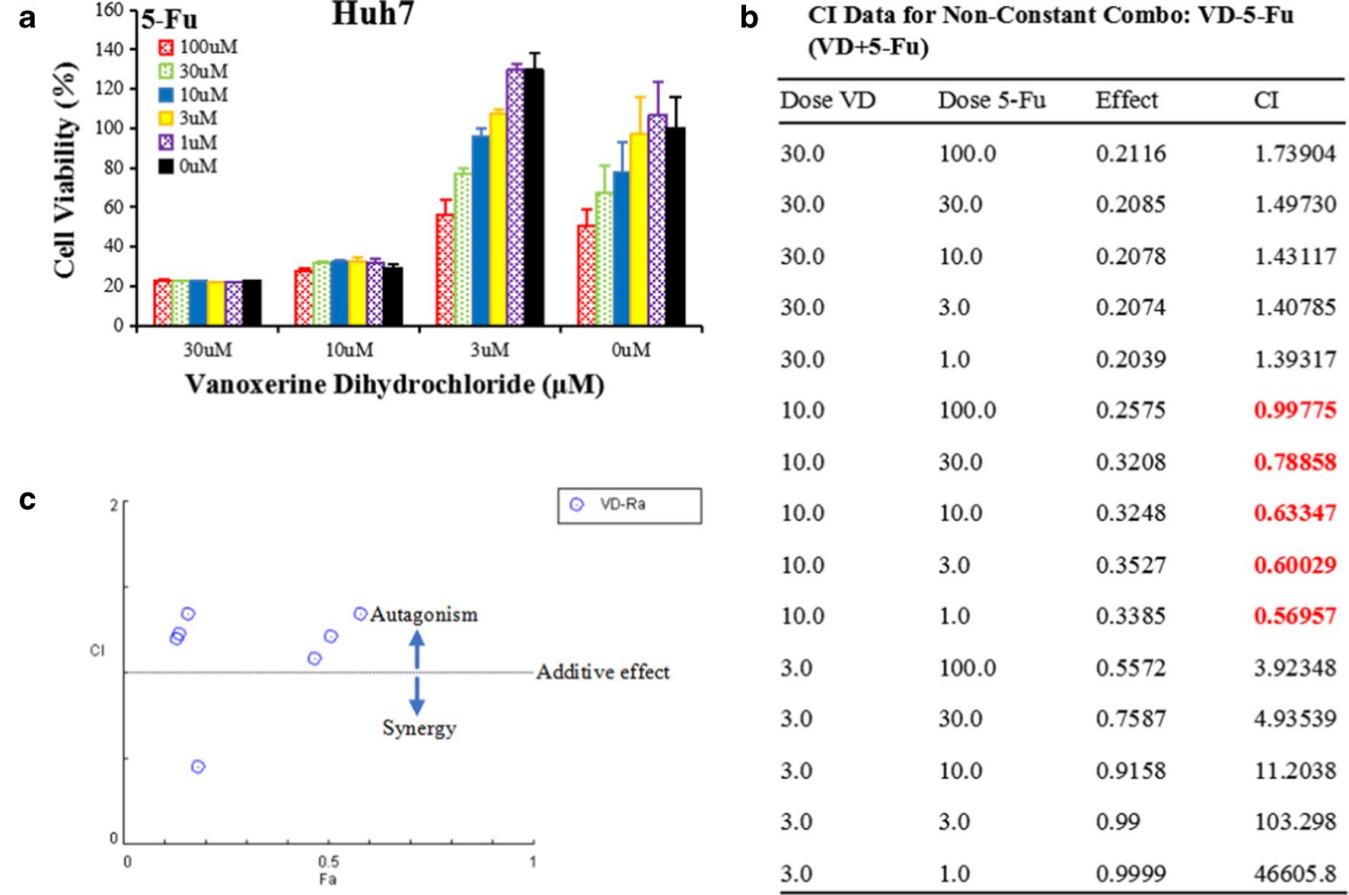
Combination of vanoxerinedihydrochloride and 5-FU produced synergistic cytotoxic effects in Huh7 cells. Huh7 cells were seeded in 96-well plates, and treated with indicated concentrations of vanoxerine dihydrochloride and 5-Fu. **a** Cell viability was detected by CCK8 after 72 h treatment. **b** The combined effect was analyzed by CompuSyn software analysis of the Combination Index (CI) of the combined action. **c** A dot plot of the combined action of vanoxerine dihydrochloride and 5-Fu

To test the potential synergistic effect of combination therapy, Huh7 cells were seeded in 96-well plates and treated with combinations of various concentrations of vanoxerine dihydrochloride (3 μM, 10 μM, 30 μM) and 5-Fu (1 μM, 3 μM, 10 μM, 30 μM, 100 μM). Cell viability was determined by CCK8 assay at 72 h after treatment (Fig. 7a, b). The drug combination effect and the combination index (CI) were analyzed by CompuSyn software to calculate the multiple drug dose–effect using the Median Effects methods described by Chou and Talalay. The quantitative definition of drug combinations is CI = 1 for additive effect, CI < 1 for synergism, and CI > 1 for antagonism. The combination of vanoxerine hydrochloride 10 μM, and 5-Fu 1 μM, 3 μM, 30 μM, 100 μM, all showed combined synergistic effect (CI < 1). CI were also used in the combined action point diagram (Fig. 7c) to quantitatively describe the synergism and antagonism of combined drugs at a given dose–effect level.

### Vanoxerinedihydrochloride administration reduced the growth of xenograftedHuh7 tumorsin vivo in nude mice

**Fig. 8:**
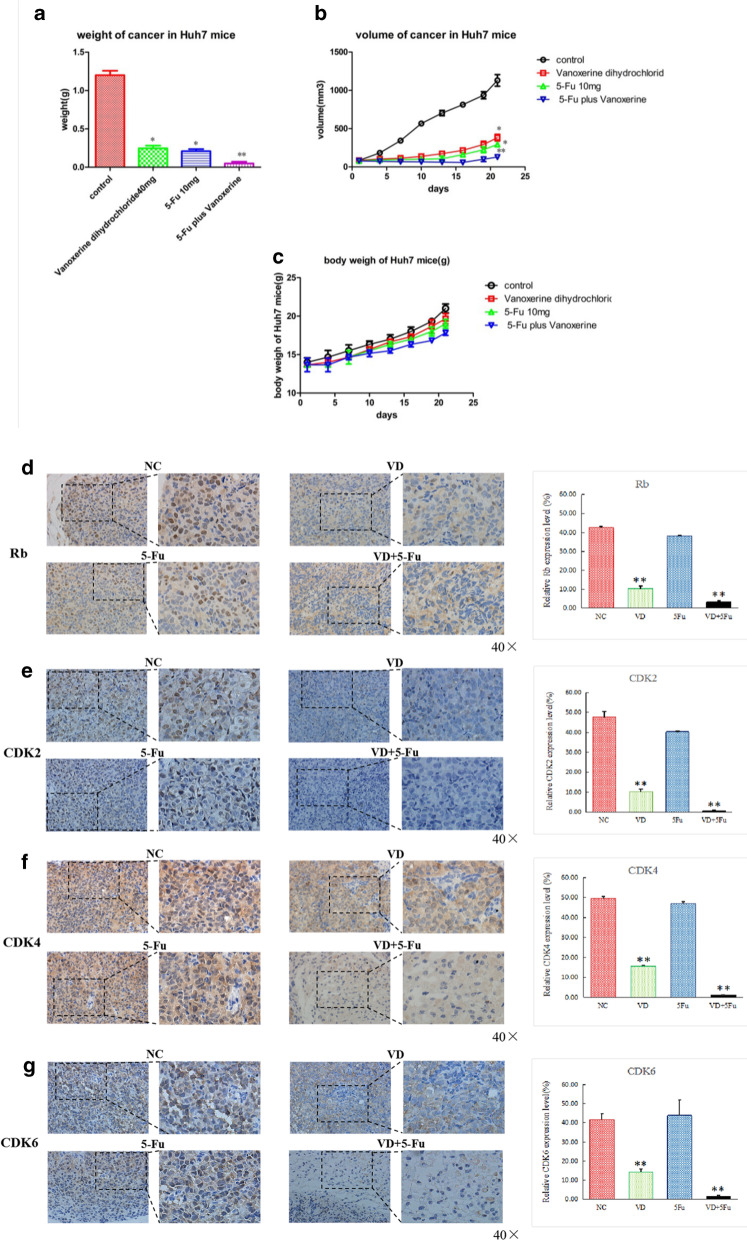
Vanoxerine dihydrochloride and 5-FU treatments reduced tumor growth in vivo in nude mice xenografted with Huh7 cells. BALB/C nude mice xenografted with Huh7 cells were treated with vanoxerine dihydrochloride (40 mg/kg), 5-Fu (10 mg/kg), vanoxerine dihydrochloride (40 mg/kg) plus 5-Fu (10 mg/kg), and PBS for 21 days by daily i.p. injections. **a** Tumor volumes. **b** Tumor weight as compared to control at day 21 after treatment. **c** Body weight. **d**–**g** The representative pictures of immunohistochemistry staining of the xenografted tumor tissues for **d** Rb, **e** CDK2, **f** CDK4, and **g** CDK6 expressions. **p < 0.01, significantly different from the control PBS treatment group

Huh7 cells (1 × 10^6^ cells in 0.2 ml PBS) were subcutaneously injected into the right flank of BALB/C nude mice. When the tumors grew to 80–100 m^3^ (7 days after inoculation), mice were divided randomly into 4 groups (5 mice/group),and treated daily for 21 days by i.p. injection of (1) control PBS, (2) vanoxerinedihydrochloride (40 mg/kg), (3) 5-Fu (10 mg/kg), (4) vanoxerinedihydrochloride (40 mg/kg) plus 5-Fu (10 mg/kg), and the tumor volume and body weight were recorded daily. At the end of experiments, mice were sacrificed by cervical dislocation. The tumor tissues were excised, weighed, images captured, and immunohistochemistry analysis performed.Vanoxerinedihydrochloride and 5-FU treatments both significantly reduced tumor weight (Fig. 8a) and tumor volume (Fig. 8b), with comparable efficacy, and the combination of vanoxerinedihydrochloride and 5-FU produced the strongest therapeutic effect. As shown in Fig. 8c, all treatments had no obvious effect on body weight. Immunohistochemistry staining of the tumor tissues showed significantly reduced expressions of Rb (Fig. 8d), CDK2 (Fig. 8e), CDK4 (Fig. 8f), and CDK6 (Fig. 8g) in vanoxerinedihydrochloride treatment group, as compared to control PBS treatment group. In contrast, 5-Fu did not show significant effect. Furthermore, the combination of vanoxerinedihydrochloride and 5-FU appeared to further decrease the expressions of these proteins.

## Discussions

In recent years, a large number of CDK inhibitors have been reported. The first generation inhibitors flavopiridol, (R)-roscovitine, and olomoucine, had low individual CDK specificity, low therapeutic efficacy and high toxicity (Senderowicz 1999; Kaur et al. 1992). The second generation of CDK inhibitors including dinaciclib, AT7519, milciclib, TG02, CYC065 and RGB-286638 demonstrated little clinical activity (Parry et al. 2010; Gojo et al. 2013; Stephenson et al. 2014; Lao et al. 2012; Mita et al. 2014). In 2015, a selective CDK 4/6 inhibitor palbociclib was approved by FDA as the first CDK inhibitor the treatment of breast cancer (Otto and Sicinski 2017). However, so far, no CDK inhibitor has been approved for the treatment of HCC or other cancers, suggesting the need to find more effective drugs, and a CDK2/4/6 triple inhibitor may be a potential candidate.

In this study, we used computer-aided strategy to screen for CDK2/4/6 triple inhibitors, and successfully discovered vanoxerine dihydrochloride. We propose that a CDK2/4/6 triple-inhibitor may offer some advantages over CDK4/6 dual-inhibitor in providing broader patient selection, higher efficacy, and broader types of cancers for treatment. Firstly, CDK2, CDK4, and CDK6, these three CDKs are often all elevated in clinical patient samples of many cancers. In HCC, CDK2, CDK4, and CDK6 have been shown to be elevated in 84% (Li et al. 2002), 66.7% (Kim et al. 2000), and 46% (Che et al. 2012) of clinical patient samples, respectively. In lung cancers, CDK2 levels were over-expressed in more than 90% (Kawana et al. 1998), and CDK4/6 in more than 23% of the patient samples (Wikman et al. 2005; Weir et al. 2007) Therefore, a CDK 2/4/6 triple inhibitor will likely be more effective than CDK4/6 dual inhibitor in these cancers.

In addition, CDK2 has different and broader functions than CDK4/6. The CDK4/6 promote cell cycle G1-S phase transition through activation of cyclinD-CDK4/6 complexes (Asghar et al. 2015; Malumbres and Barbacid 2009), hyper-phosphorylation ofRbon serine and threonine residues. (Cobrinik 2005), and stimulation ofthe release of E2F transcription factor, which facilitates the transcription of genes required for G1-to-S transition and S-phase progression. CDK2 works differently. It promotes G1-S phase transition through activation of cyclin E-CDK2 to maintain Rb phosphorylation. It also activates cyclinA-CDK2 complexes, to initiate DNA synthesis and the S phase cell cycle, and cyclin A1 has been reported to be over-expressed with highest expression at the preneoplastic stage in human HCC. HBV and HCV are two major risk factors for liver cirrhosis and HCC. Computational analysis in the protein–protein interaction network of HBV proteins has identified not only CDK4/ 6 but also CDK2 as HCC-related genes (Jiang et al. 2013), and interaction network of HCV proteins has identified CDKN2A (cyclin-dependent kinase inhibitor 2A) as one of the HCC related overlapped genes (Huang et al. 2012). Emerging evidence has strongly suggested that CDK2/4/6, in particular the CDK2, are involved in RNA modifications. As m6A RNA methylation participates in the pathogenesis of multiple diseases including cancer, the potential roles of CDK2/4/6 in m6A RNA modification in human HCC require further investigations (Li et al. 2020). Furthermore, CDK2 has been reported to phosphorylate the p27^KIP1^ and RB proteins in cell cycle progression, the replication factors A and C in DNA replication, the NPAT in histone synthesis, and the nucleophosmin (NPM) in centrosome duplication (Meraldi et al. 1999).Taken together these studies strongly suggested an important role of CDK2 in human HCC, and a CDK2/4/6 triple inhibitor, vanoxerine dihydrochloride, may have additional advantages and broader anti-cancer activities than CDK4/6 dual inhibitors for the treatment of human HCC.

We compare vanoxerine dihydrochloride with two CDK2 inhibitors, Adapaline (Shi et al. 2015a) and Fluspirilene (Shi et al. 2015b), and one CDK4/6 dual inhibitor Rafoxanide (Shi et al. 2018) we identified from FDA approved drugs by similar strategies. Vanoxerine dihydrochloride has similar anti-cancer activities in inhibiting cell growth, with IC50 equal to 3.79 μM in QGY7703 and 4.04 μM in Huh7 cells. The other three compounds also have similar IC50 values (IC50 for fluspirilene is 4.01 μM in HepG2 and 3.46 μM in Huh7 cells; for adapalene is 4.43 µM in DLD1 and 7.135 µM in LoVo cells, andfor rafoxanide in skin cancer is 1.09 µM in A375 cells and 1.31 µM in A431 cells).As CDK inhibitors, they all have the abilities to inhibit cell cycle progression, and induce apoptosis in cell culture models. They all are capable of reducing tumor growth in vivo in nude mice xenograted preclinical animal models. However, as a CDK2/4/6 triple inhibitor, vanoxerine dihydrochloride may have broader activity and will be effective to a larger number of cancers than CDK2 or CDK4/6 inhibitors. In addition, these compounds are different in their physical and chemical properties. Therefore, they require different drug delivery systems, suitable for the treatment of different type of cancers, and also have different side effects.

Wealso compared vanoxerine dihydrochloride with Palbociclib (Hsieh et al. 2017) in terms of efficacy, cell toxicity, and animal toxicity for the treatment of HCC. In the in vivo nude mice xenografted preclinical HCC animal models, vanoxerine dihydrochloride (i.p. 40 mg/kg per day for 21 days) and Palbociclib (orally 150 mg/kg every three days for 18 days) both reduced tumor growth significantly. Vanoxerine dihydrochloride treatment did not caused significant change in body weight, while Palbociclib treatment produced a slight loss of body weight. In the in vitro cell culture studies, vanoxerine dihydrochloride had similar or higher cell cytotoxicity than Palbociclib in the HCC cells tested. For examples, the calculated IC50 for vanoxerine dihydrochloride was 3.79 μM in QGY7703 and 4.04 μM in Huh7 cells, while the reported IC50 for Palbociclib was 5 μM in Hep3B, 10-15μMin Huh7 and > 25 μM in PLC5 cells. These results suggested that Palbociclib and vanoxerine dihydrochloride have comparable efficacy and toxicity for HCC treatment.

Furthermore, we demonstrated the synergic effect of combining vanoxerine dihydrochloride with chemotherapy drug 5-Fu both in vitro in cell lines and in vivo in preclinical animal models. The combination therapies have already been shown to be beneficial for CDK4/6 dual inhibitors. For examples, FDA has approved the use of palbociclib in combination with fulvestrant for the treatment of hormone receptor-positive, HER2-negative metastatic breast cancer (Walker et al. 2016). The potential additive or synergistic effect of combination therapy of vanoxerine dihydrochloride with other targeted therapies, chemotherapies, radiotherapy or immunotherapies warrant further investigations.

Vanoxerine was originally developed as a dopamine transporter antagonist for the treatment of depression and Parkinson’s disease, but later failed todemonstrate significant benefit for these diseases (Lane et al. 2005; Spealman et al. 1989; Howell and Byrd 1991; Howell and Landrum 1997; Nagase et al. 1991; Giros et al. 1992). The safety of vanoxerine dihydrochloride in animals have been reported by Nagase and coworkers (Nagase et al. 1987b). They showed that oral administration of vanoxerine (50–250 mg/kg) to male rats produced a transient increase in dopamine content of the caudate nucleus and hypothalamus, and a slight decrease of norepinephrine levels in the hypothalamus and frontal cortex. In addition, administration (20 mg/kg, i.p.) of vanoxerine caused marked increase in locomotor activity (Heikkila and Manzino 1984).In MES test (a model for generalized tonic–clonic seizures), intraperitoneal administration of vanoxerine at 80 mg/kg and above, produced toxicity with an inability to grasp a rotorod, muscle spasms, minimal motor impairment in mice (Goldsmith et al. 2007). The calculated TD50 (i.p.) for vanoxerinewas 77.5 mg/kg in mice and 74 mg/kg in rats.In preclinical studies in nonhuman primates, the reported LD50 (oral) is 500 mg/kg (Nagase et al. 1987a; Howell and Landrum 1997), and no obvious toxic effects were reported following intraperitoneal (i.p.) injection at 20 mg/kg (Howell and Landrum 1997). In the present study we did not observe any significant changes in the body weight of the BALB/C nude mice administered (i.p.) withvanoxerinedihydrochloride (40 mg/kg) over 21 days.

Vanoxerine was later found to have desirable cardiac antiarrhythmic properties (Matsumoto et al. 2010).Patch clamp studies showed that it potently blocked IKr (hERG), L-type calcium and sodium channels (Preti 2000).In a phase II dose-ranging COR-ART study, vanoxerine was highly effective in converting atrial fibrillation and atrial flutter (AF/AFL) to sinus rhythm without evidence of proarrhythmia (Howard et al. 2015). However, this drug was eventually terminated from development due to occurrence of cardiac arrhythmias including torsade de pointes (TdP) in patients with structural heart disease (Piccini et al. 2016).

To the best of our knowledge, the present study was the first to report that vanoxerine dihydrochloride is a CDK2/4/6 triple inhibitor, and that vanoxerine dihydrochloride exhibited significant in vivo anti-cancer efficacy. As a FDA approved drug, the use of vanoxerine dihydrochloride for the treatment of HCC and other cancers warrant further investigations.

## Conclusion

In this study, we reported the discovery of a new CDK2/4/6 triple inhibitor, vanoxerine dihydrochloride. Due to the important roles of CDK2 in HCC, a CDK2/4/6 triple inhibitor may have additional advantages and broader anti-cancer activities than CDK4/6 dual inhibitors for the treatment of human HCC and other cancers.

## Data Availability

ALL of the data generated or analysed during this study are included in this published article.

## Abbreviations

CDK: Cyclin-dependent kinases
HCC: Hepatocellular carcinoma
FDA: Food and drugs administration
Rb: Retinoblastoma protein
TKI: Tyrosine kinase inhibitor
5-Fu: 5-Fluorouracil
CDKN2A: Cyclin-dependent kinase inhibitor 2A
IC50: Half maximal inhibitory concentration
TD50: Median toxic dose
LD50: Median lethal dose
